# Operative treatment outcomes of anterior sternoclavicular joint dislocation using two experimental methods - an acromioclavicular joint hook plate versus a locking plate: a retrospective study

**DOI:** 10.1186/s12891-022-05293-x

**Published:** 2022-04-11

**Authors:** Yanzhen Qu, Xudong Xie, Wu Zhou, Tian Xia, Faqi Cao, Bobin Mi, Yuan Xiong, Zhewei Ye, Guohui Liu

**Affiliations:** Department of Orthopedics, Union Hospital, Tongji Medical College, Huazhong University of Science and Technology, 430022 Wuhan, People’s Republic of China

**Keywords:** Sternoclavicular joint dislocation, Acromioclavicular joint hook plate, Locking plate, Physical function

## Abstract

**Background:**

We aimed to compare the intraoperative and early postoperative clinical outcomes of using an acromioclavicular joint hook plate (AJHP) versus a locking plate (LP) in the treatment of anterior sternoclavicular joint dislocation.

**Methods:**

Seventeen patients with anterior sternoclavicular joint dislocation were retrospectively analyzed from May 2014 to September 2019. Six patients were surgically treated with an AJHP, and 11 were surgically treated with an LP. Five male and one female patients composed the AJHP group, and nine male and two female patients composed the LP group. The mean age of all patients was 49.5 years.

**Results:**

Reduction and fixation were performed with AJHP or LP in all 17 patients. The mean operative blood loss, operative time, and length of incision in the AJHP group were significantly better than those in the LP group. Shoulder girdle movement of the AJHP group was significantly better than that of the LP group.

**Conclusions:**

This study revealed that AJHP facilitated glenohumeral joint motion, reduced the risk of rupture of mediastinal structures, required a shorter incision, and had lesser blood loss and a shorter duration of operation compared with LP. However, some deficiencies require further improvement.

**Supplementary information:**

The online version contains supplementary material available at 10.1186/s12891-022-05293-x.

## Background

The sternoclavicular joint is the only bony articulation between the upper extremity and axial skeleton [1–3]. The ligamentous structures surrounding the sternoclavicular joint maintain its stability and make it a constricted joint [4]. Due to the presence of these stable structures surrounding the sternoclavicular joint, sternoclavicular joint dislocation is infrequent, representing only 3% of all dislocations in the shoulder girdle treated clinically [5, 6]. Dislocation can be divided into anterior dislocation and posterior dislocation by different injury mechanisms, dislocation directions, and clinical manifestations. The incidence of anterior dislocation is approximately 90% of all sternoclavicular joint dislocation [7]. Lateral compressive force on the shoulder girdle can cause the anterior capsule and costoclavicular ligament to rupture, which results in anterior dislocation [4]. These damaged structures make the joint unstable, and manual reduction is difficult to maintain, leading to frequent cases of re-dislocation [3, 4]. Therefore, surgery is recommended for very disabling instability in order to reduce the occurrence of unstable dislocation, but less disabling instability can be managed non-operatively.

There are many different surgical methods that have been designed for the treatment of anterior sternoclavicular joint dislocation, such as Kirschner wires, FiberWire, two screws and a strong suture, T-plate, locking plate (LP), and acromioclavicular joint hook plate (AJHP) [8–13]. Regardless of the surgical methods performed, important thoracic structures which exist behind the sternoclavicular joint, such as the trachea, esophagus, brachiocephalic vein, brachiocephalic artery, and brachial plexus, were protected from accidental injury caused by surgical treatment, especially on the sternal manubrium [3, 14, 15]. As a classical surgical method, LPs have been used for the reduction and fixation of the sternoclavicular joint [16, 17]. Additionally, we also have reported the safety and efficacy of using an AJHP for the treatment of anterior sternoclavicular joint dislocation [13]. However, studies evaluating the intraoperative and early postoperative clinical outcomes of AJHP compared with other kinds of internal fixation are lacking.

This study aimed to compare the intraoperative and early postoperative clinical outcomes of using an AJHP to LPs in a series of 17 patients who were followed up for a minimum of 3 months.

## Methods

The Ethics Committee of Tongji Medical College, Huazhong University of Science and Technology approved this study.

### General data

From May 2014 to September 2019, 17 patients with anterior sternoclavicular joint dislocation were enrolled into our study. Of these, six patients were surgically treated with an AJHP, and 11 were surgically treated with an LP.

The exclusion criteria were as follows: (1) patients with brain injury or other serious illnesses that increased their risk throughout surgery or anesthesia; (2) patients who demanded conservative treatment even if the closed reduction was useless; (3) patients with spinal cord injury or peripheral nerve injury that affected upper limb movement; and (4) patients with fracture, scapulohumeral periarthritis, or rotator cuff tear at the same side of the upper limb with movement difficulties. There were five male and one female patients in the AJHP group and nine male and two female patients in the LP group. The mean age of all patients was 49.5 years. One patient in the AJHP group experienced bilateral dislocation of the sternoclavicular joints. Four patients had rib fractures, one had an ankle fracture, one had a lumbar vertebra fracture without spinal cord injury, and one had a thoracic vertebra fracture without spinal cord injury. The mechanisms of injury that caused the dislocation varied. Nine patients were injured in car accidents, five experienced accidental falls from heights, two were injured by falling from driving a motorbike, and one was injured by beating. All patients were immobilized with a sling for the affected side shoulder, and underwent the standard preoperative assessment, including preoperative history, physical examination, radiography, and computed tomography. Closed reduction was attempted for all patients but was found to be unproductive, and surgery was chosen as the best mode of treatment. The interval between injury and surgery ranged from 1 to 15 days.

### Surgical technique

**Fig. 1:**
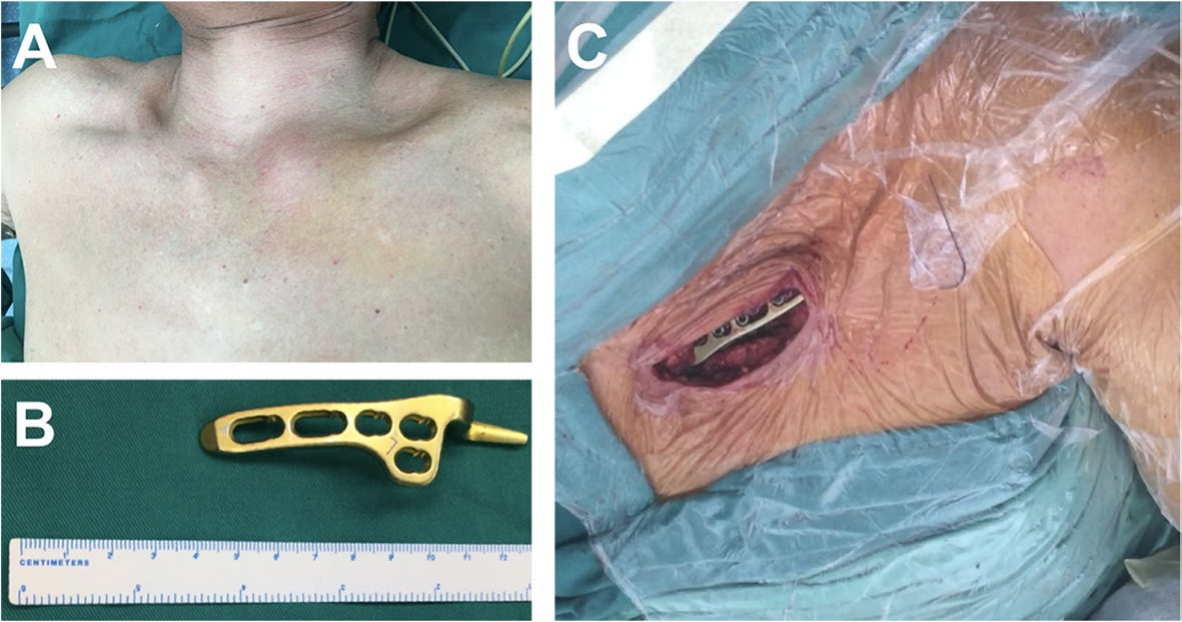
**A** The locatl bump in the left medial clavicle indicates the anterior sternoclavicular joint dislocation. **B** The acromioclavicular joint hook plate (AJHP) used in the surgical treatment for anterior sternoclavicular joint dislocation. **C** The surgical incision and status of acromioclavicular joint hook plate (AJHP) fixation

All patients were placed in the supine position on the operating table and were administered with general anesthesia. For patients who underwent AJHP surgery, an anterosuperior straight incision was made, extending from the medial part of the clavicle to the middle of the superior aspect of the sternal manubrium, and the sternoclavicular joint, sternal manubrium, and medial clavicle were exposed (Fig. 1). The incarcerated soft tissue of the sternoclavicular joint was cleaned, and the broken sternoclavicular joint cartilage plate was replaced or cleaned. The pointed end of the AJHP was inserted into the dorsal osteal surface of the sternal manubrium, and the lever effect was used to press the proximal end of the clavicle down to achieve reduction. When reduction was successfully performed, three or four bicortical screws were inserted into the clavicle. The broken anterior sternoclavicular and costoclavicular ligaments were repaired using absorbable sutures.

For patients who underwent LP surgery, an anterosuperior curved incision was made extending from the medial part of the clavicle to the upper part of the mesosternum. After the incarcerated soft tissue and broken sternoclavicular joint cartilage plate were cleaned, the proximal end was pressed to reduce the dislocation, and a Kirschner wire was used for temporary fixation. The LP was bent and placed on the surface of the clavicle and sternum, 3–4 bicortical screws were inserted into the clavicle, and 3–4 unicortical screws were fixed into the sternum. The broken ligaments were then repaired.

### Postoperative management

In the first 4 weeks postoperatively, the shoulder was immobilized with a sling for both groups, and easy exercises of the glenohumeral joint in the range of 0°–90° abduction were authorized. After 4 weeks, the range of motion was increased in accordance with each patient’s healing course. Within the first 3 months, it was recommended that sporting activities be avoided.

### Follow-up

All patients were followed up for a mean duration of 14.4 (range, 3–28) months. Radiography and range of motion measurement of the glenohumeral joint were performed on the first postoperative day; after 4, 8, and 12 weeks; and then once every 6 months.

### Statistical analysis

Data were exhibited as means ± standard deviations. All data were tested for normal distribution using the Kolmogorov–Smirnov test. Then, the data of the two groups were compared using the nonparametric Mann–Whitney U-test for independent samples and Student’s t-test for dependent samples. Differences were considered significant with *P* < 0.05.

## Results

### General data

**Fig. 2:**
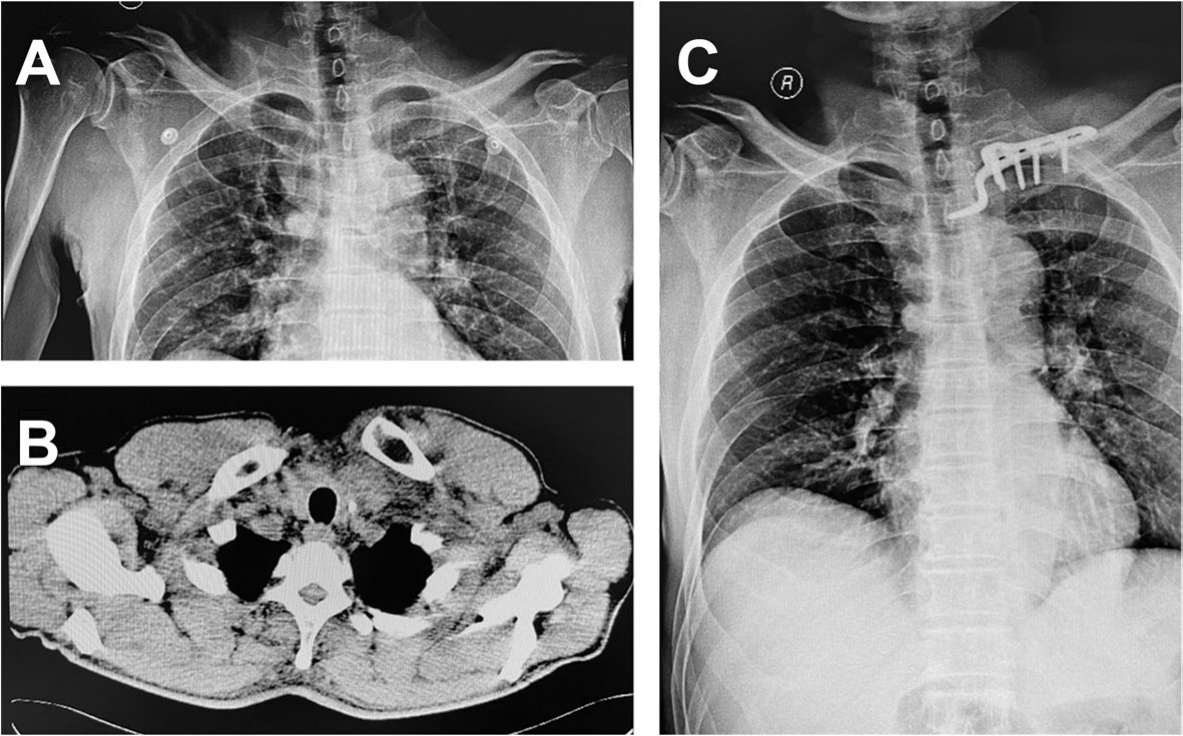
Left anterior sternoclavicular joint dislocation treated with an acromioclavicular joint hook plate (AJHP). **A **The preoperative radiograph of the patient. **B** The preoperative computed tomography (CT) scan shows dislocation of the left anterior sternoclavicular joint. **C** The postoperative radiograph of the patient shows good reduction of the left sternoclavicular joint

In the AJHP group, one patient underwent bilateral operation, five underwent unilateral operations, and a total of seven sternoclavicular joints underwent surgery (Fig. 2). For the seven joint operations, the mean operative blood loss of each joint was 52.9 (range, 35–80) mL, and the mean operative time was 0.73 (range, 0.5–1.08) h. The mean incision length was 8.21 (range, 6.5–9.5) cm.

**Fig. 3:**
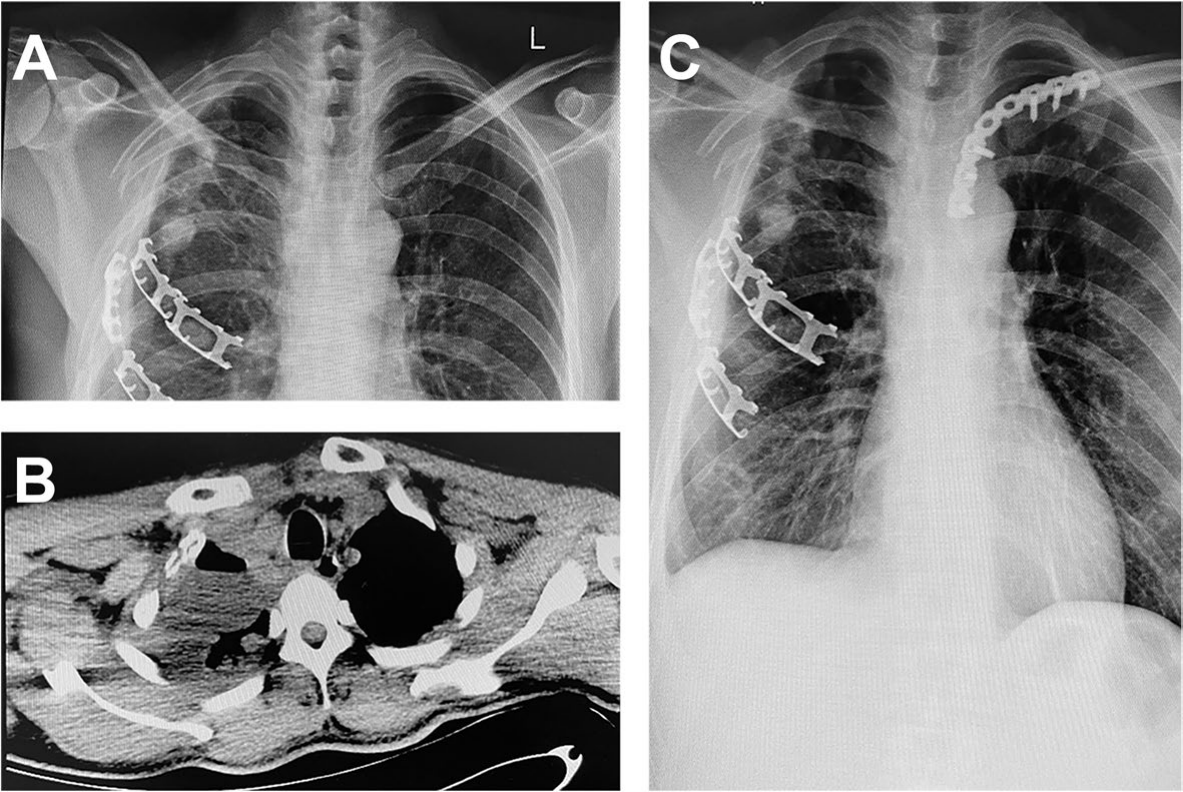
Left anterior sternoclavicular joint dislocation treated with a locking plate (LP). **A **Preoperative radiograph of the patient. **B** Preoperative computed tomography (CT) scan shows a dislocation of the left anterior sternoclavicular joint. **C** Postoperative radiograph of the patient shows good reduction of the left sternoclavicular joint

In the LP group, 11 patients underwent unilateral operations (Fig. 3). For the 11 joint operations, the mean operative blood loss of each joint was 83.2 (range, 60–150) mL, and the mean operative time was 1.68 (range, 0.83–2.25) h. The mean incision length was 17.5 (range, 15–20) cm.

There were no respiratory or circulatory abnormalities reported in any of the patients considered. No postoperative incision infection and no complications, such as joint re-dislocation, vascular rupture, or vital organ injury, were observed. One patient in the LP group had pneumothorax after the operation, but the patient also underwent same-side rib fracture internal fixation in the same operation. Associated injuries were treated effectively. There was no plate breakage or screw breakage reported at the final follow-up.

### Radiography, motion range measurement, and physical function

**Fig. 4:**
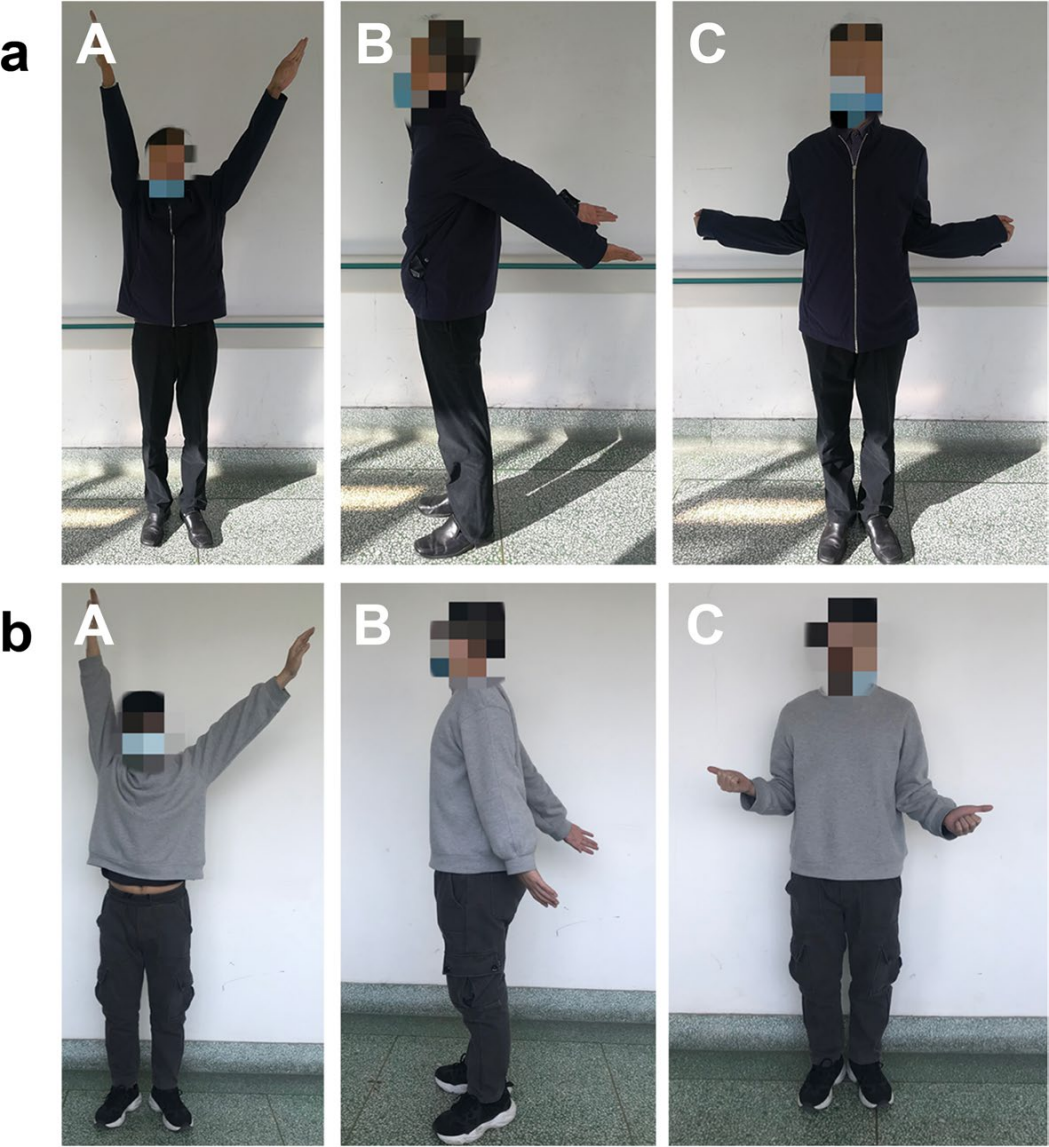
**a** Figure shows the postoperative glenohumeral joint function of a patient at 1 year and 2 months after a left anterior sternoclavicular joint dislocation that was treated with an acromioclavicular joint hook plate (AJHP). **b** Figure shows the postoperative glenohumeral joint function of a patient at 1 year and 6 months after sustaining a left anterior sternoclavicular joint dislocation that was treated with a locking plate (LP). **A** Abduction of the glenohumeral joint. **B** Posterior extension function of the glenohumeral joint. **C** External rotation of the glenohumeral joint

Postoperative radiography showed that all dislocated joints were successfully treated, and the location and angle of the plates were suitable. No re-dislocation occurred during the period of follow-up. The mean postoperative abduction angle, the mean posterior extension angle, and the mean external rotation angle of glenohumeral joint in both groups were described in Table 1. The shoulder girdle movement of patients in the AJHP group was significantly better than that of patients in the LP group (Fig. 4a).

**Table 1:** Operation-related information and postoperative evaluation data

Variables	Groups		p*
	AJHP	LP	
Blood Loss (ml)	52.8571 ± 15.50729	83.1818 ± 24.92717	0.011
Operative Time (hour)	0.7257 ± 0.18946	1.6809 ± 0.38006	0.000
Incision Length (cm)	8.2143 ± 1.11270	17.5455 ± 1.63485	0.000
Abduction Angle (°)	164.2857 ± 8.86405	146.8182 ± 11.46140	0.004
Posterior Extension Angle (°)	45.7143 ± 5.34522	32.7273 ± 8.47456	0.002
External Rotation Angle (°)	71.4286 ± 10.29332	57.7273 ± 6.84238	0.004

* Independent samples *t* test

## Discussion

The ligaments that surround the sternoclavicular joint guarantee its stability; however, the joint is inherently unstable [2, 3]. A direct force on the medial part of the clavicle could lead to posterior dislocation of the joint [15]. Indirect lateral compressive forces that affect the shoulder could rupture the anterior capsule and ligaments via the lever effect of the clavicle and can potentially result in anterior sternoclavicular joint dislocation [7, 15]. Closed reduction is the first choice for the treatment of anterior sternoclavicular joint dislocation, but it is usually unsatisfactory due to ligamentous rupture, which makes the joint unstable [3]. The sternoclavicular joint is the only bony articulation between the axial skeleton and upper extremity, and movement at the joint, which is produced by the transmission of the movements of the scapula on the chest wall, can occur passively in three planes [1, 15]. During shoulder abduction, the sternoclavicular joint can move in the coronal and anteroposterior planes. Therefore, preservation of micromotion in the sternoclavicular joint has a significant effect on the postoperative range of glenohumeral joint motion [18]. According to our research, patients in the LP group had limited movement of the sternoclavicular joint; therefore, glenohumeral joint motion was significantly limited relative to that of those in the AJHP group.

There are many important thoracic structures are located posterior to the sternoclavicular joint [14, 15]. The fact that these complex structures surround the sternoclavicular joint implies that dislocation could cause serious trauma, such as rupture of blood vessels, nerve injury, pleura rupture, lung rupture, and mediastinum organ injury. Additionally, operations on the joint require increased caution. In the LP group, drilling and screwing on the sternum could increase the risk of rupture or injury to mediastinal structures, although such complications were not observed in this study.

Comparing the incisions used to surgically treat both groups, in the AJHP group, a small part of the sternal manubrium was exposed, which was sufficient for plate insertion. However, in the LP group, most parts of the sternal manubrium, and even the mesosternum, was exposed to facilitate fixation of the plate and screws. Due to the shorter incision and smaller number of screws needed, blood loss in the AJHP group was significantly lower than that in the LP group. In addition, LP bending, drilling, and screwing on the sternum prolonged the duration of the operation in the LP group.

In our research, AJHP and LP were used for the treatment of sternoclavicular joint dislocation. Some advantages of AJHP treatment were as follows: (1) AJHP facilitates micromotion within a certain range between the hook and manubrium of the sternum, which is beneficial for glenohumeral joint motion; (2) the hook structure behind the sternal manubrium could reduce the risk of mediastinal structure rupture during drilling and screwing; and (3) the AJHP operation method requires a shorter incision, produces less blood loss, and has a shorter operation time.

However, there are some disadvantages of this study, such as the following: (1) the structure of the AJHP is not suitable for the anatomical structure of the sternoclavicular joint, and the plate is not in line with the joint; (2) there is no special screw hole available for sternoclavicular joint dislocation with medial clavicle fracture; (3) for treatment with LP, the repetitive stress of joint micromotion may cause loosen or break of the plate, so the LP should be removed early but most of them were removed with 1 year or longer after surgery in our study; (4) compared with LP, the hook structure of AJHP facilitates micromotion within a certain range, and reduces the probability of plate loosen or break. However, risk of hook cutting through the sternum still exists due to the sharpness of the hook of the plate when the AJHP is used to treat patients with anterior sternoclavicular joint dislocation although the case has not been found in our study; and (5) AJHP might provide good treatment outcomes in patients with anterior sternoclavicular joint dislocation in this study, but it might carry risks to penetrate the mediastinal structures in patients with posterior dislocation of sternoclavicular joint. (6) there is still no guideline for the treatment of sternoclavicular joint dislocation, the treatments of AJHP and LP are experimental, and patients should be informed the disadvantages of using AJHP or LP clearly, such as cutting through the sternum, vascular injury and reoperation [19]. Based on these disadvantages, the current AJHP needs to be promoted in these details in the future, for example, the time of removing AJHP.

## Conclusions

A comparison of AJHP and LP revealed that AJHP provided increased glenohumeral joint motion, reduced the risk of rupture of mediastinal structures, required a shorter incision, incurred less blood loss, and required a shorter operation time. However, AJHP still has some disadvantages. Further studies are needed to improve the design of the new hook plate in the future.

## Supplementary information

**Additional file 1.** Source data patient information.

## Data Availability

The datasets used and/or analyzed during the current study are available from the corresponding author on reasonable request.

## Abbreviations

AJHP: Acromioclavicular joint hook plate
LP: Locking plate
CT: Computed tomography
