# Supplementary material for: Operative treatment outcomes of anterior sternoclavicular joint dislocation using two experimental methods - an acromioclavicular joint hook plate versus a locking plate: a retrospective study
Source: BMC Musculoskelet Disord. 2022 Apr 11;23:350. doi: 10.1186/s12891-022-05293-x (PMC8996669; doi:10.1186/s12891-022-05293-x)
Supplement: Supplementary file 1 — Additional file 1. Source data patient information. [file 12891_2022_5293_MOESM1_ESM.doc]

**Source data: patient information**

| **Group** | **Age** | **Sex** | **Follow-up (months)** | **Mechanisms of injury** | **Blood loss(ml）** | **Operative time(h)** | **Incision length(cm)** | **Abduction angle(°)** | **Posterior extension angle(°)** | **External rotation angle(°)** |
| --- | --- | --- | --- | --- | --- | --- | --- | --- | --- | --- |
| **AJHP** | **49** | **Male** | **22** | **Car accident** | **45** | **0.75** | **7** | **165** | **45** | **70** |
|  |  |  |  |  | **35** | **0.5** | **6.5** | **155** | **40** | **70** |
| **AJHP** | **64** | **Male** | **18** | **Car accident** | **80** | **1.08** | **9.5** | **175** | **45** | **50** |
| **AJHP** | **53** | **Male** | **26** | **Car accident** | **65** | **0.67** | **8** | **170** | **45** | **80** |
| **AJHP** | **44** | **Male** | **7** | **Car accident** | **55** | **0.67** | **8.5** | **150** | **55** | **80** |
| **AJHP** | **51** | **Male** | **13** | **Fall from height** | **50** | **0.58** | **9** | **165** | **50** | **75** |
| **AJHP** | **42** | **Female** | **20** | **Car accident** | **40** | **0.83** | **9** | **170** | **40** | **75** |
| **Mean** |  |  |  |  | **52.857142** | **0.7257142** | **8.2142857** | **164.285714** | **45.7142857** | **71.428571** |
|  |  |  |  |  |  |  |  |  |  |  |
| **Group** | **Age** | **Sex** | **Follow-up (months)** | **Mechanisms of injury** | **Blood loss(ml）** | **Operative time(h)** | **Incision length(cm)** | **Abduction angle(°)** | **Posterior extension angle(°)** | **External rotation angle(°)** |
| **LP** | **46** | **Male** | **28** | **Car accident** | **60** | **0.83** | **15** | **160** | **50** | **70** |
| **LP** | **20** | **Male** | **13** | **Fall from height** | **90** | **1.67** | **18** | **130** | **20** | **45** |
| **LP** | **48** | **Male** | **11** | **Fall from height** | **90** | **1.83** | **18** | **150** | **35** | **60** |
| **LP** | **54** | **male** | **14** | **Beating** | **70** | **2** | **20** | **140** | **40** | **60** |
| **LP** | **42** | **Female** | **11** | **Car accident** | **150** | **2.25** | **20** | **130** | **35** | **50** |
| **LP** | **61** | **Male** | **13** | **Fall from height** | **90** | **1.5** | **18** | **160** | **40** | **60** |
| **LP** | **48** | **Male** | **7** | **Car accident** | **70** | **1.75** | **18** | **140** | **30** | **55** |
| **LP** | **63** | **Male** | **14** | **Driving a motorbike** | **90** | **1.25** | **16** | **140** | **25** | **55** |
| **LP** | **51** | **Female** | **12** | **Driving a motorbike** | **70** | **1.83** | **16** | **160** | **25** | **65** |
| **LP** | **50** | **Male** | **13** | **Car accident** | **70** | **1.75** | **16** | **150** | **30** | **60** |
| **LP** | **55** | **Male** | **3** | **Fall from height** | **65** | **1.83** | **18** | **155** | **30** | **55** |
| **Mean** | **49.47058** |  | **14.41176** |  | **83.181818** | **1.6809090** | **17.545454** | **146.818181** | **32.7272727** | **57.727272** |
